# Dysregulated *miR-183 *inhibits migration in breast cancer cells

**DOI:** 10.1186/1471-2407-10-502

**Published:** 2010-09-21

**Authors:** Aoife J Lowery, Nicola Miller, Roisin M Dwyer, Michael J Kerin

**Affiliations:** 1 Department of Surgery, National University of Ireland, Galway, Ireland

## Abstract

**Background:**

The involvement of miRNAs in the regulation of fundamental cellular functions has placed them at the fore of ongoing investigations into the processes underlying carcinogenesis. MiRNA expression patterns have been shown to be dysregulated in numerous human malignancies, including breast cancer, suggesting their probable involvement as novel classes of oncogenes or tumour suppressor genes. The identification of differentially expressed miRNAs and elucidation of their functional roles may provide insight into the complex and diverse molecular mechanisms of tumorigenesis. *MiR-183 *is located on chromosome 7q32 and is part of a miRNA family which are dysregulated in numerous cancers. The aims of this study were to further examine the expression and functional role of *miR-183 *in breast cancer.

**Methods:**

*MiR-183 *expression was quantitated in primary breast tumours, tumour associated normal tissue and breast cancer cell lines using RQ-PCR. Gain of function analysis was performed in breast cancer cells using pre-miR-183 and the effect of *miR-183 *overexpression on cell viability, proliferation, apoptosis and migration was examined. Customized Taqman Low Density Arrays (TLDA) were used to identify dysregulated genes in breast cancer cells transfected with pre-miR-183.

**Results:**

We demonstrate that *miR-183 *is dysregulated in breast cancer and expression correlates with estrogen receptor and HER2/neu receptor expression. Induced overexpression of *miR-183 *inhibited migration of breast cancer cells. This finding was substantiated by RQ-PCR of mRNA from cells overexpressing *miR-183 *which showed dysregulation of several migration and invasion related genes. Specifically, the VIL2-coding protein Ezrin was confirmed as a target of *miR-183 *and downregulation of this protein was confirmed with immunocytochemistry.

**Conclusions:**

These findings indicate that *miR-183 *targets VIL2 and may play a central role in the regulation of migration and metastasis in breast cancer. Consequently, this miRNA may present an attractive target for therapeutic intervention.

## Background

Breast cancer is a heterogeneous disease and represents a number of phenotypically diverse tumours[1]. The complex nature of breast cancer makes progression difficult to predict and challenging to manage. Up to forty percent of breast cancer patients still present with regional or distant disease progression at the time of diagnosis[2], and the overall survival rates for these patients has changed very little in the past decade despite advances in systemic therapy.

The key to optimising breast cancer management lies in early detection, effective prognostication and individualised therapy, all of which demand a comprehensive understanding of the complex molecular interactions that underlie breast cancer development and progression. Gene expression profiling has gone some way towards unlocking the heterogeneity of breast cancer; the advent of such high throughout technologies as microarray expression profiling facilitated the extensive investigation of breast cancer related genes [3-5]. Breast tumors can now be classified into major subtypes on the basis of gene expression: luminal, HER2*/neu *overexpressing and basal-like, and further analysis has identified additional subtypes within the original sub-groups[6].

Multigene prognostic and predictive tests have been developed, commercialized and have become established as tools in breast cancer diagnostics[7], however as yet there is little knowledge regarding the precise regulation of these genes and receptors, and the precise manner in which they drive breast cancer progression and metastasis.

Mi(cro)RNAs represent a fascinating new division of biomolecules. The analysis of their expression will help decipher complex patterns in disease states such as malignancy, and promises to improve classification of tumors such as breast cancer [8] in addition to uncovering pathogenic pathways and therapeutic avenues.

The aim of this study was to investigate the potential role of a specific miRNA *miR-183 *in breast cancer progression. *MiR-183 *is a member of a miRNA family comprised of three homologous miRNAs (*miR-183*, *miR-182 *and *miR-96*) that are clustered within 2-4 kb at chromosome 7q32. MiRNAs from this locus are dysregulated in diverse cancers including leukaemia, hepatic, colorectal and breast cancer[9-13]. Furthermore, downregulation of *miR-183 *has been shown to be associated with metastasis in lung cancer, and its' ectopic expression inhibits the invasiveness of cancer cells [14], suggesting that *miR-183 *plays a role in carcinogenesis or the metastatic cascade, possibly having a tumour suppressor role.

In this study we investigated the expression pattern and functional role of *miR-183 *in breast cancer. This was undertaken by quantitation of *miR-183 *in breast cancer specimens in relation to known clinicopathological parameters of breast cancer. Functional analysis was then performed by transfection of *miR-183 *precursors into a breast cancer cell line with low endogenous *miR-183 *levels. The effects of transfection were subsequently assessed on cell viability patterns, cell migration and alterations in gene and protein expression by real-time quantitative PCR and immunocytochemistry respectively.

The findings of this study support a role for *miR-183 *in breast cancer, particularly in the metastatic process, and identify this miRNA as a novel therapeutic target in breast cancer.

## Methods

### Clinical Samples

Breast tumour specimens were obtained from patients during primary curative resection at Galway University Hospital, Galway, Ireland. Matched tumour-associated normal (TAN) breast tissue was also obtained from a subset of these patients where possible. Following excision, tissue samples were immediately snap-frozen in liquid nitrogen and stored at -80°C until RNA extraction. Prior written and informed consent was obtained from each patient and the study was approved by the ethics review board of Galway University Hospital. A cohort of fresh frozen breast tumor (n = 70) and TAN (n = 9) specimens was used for analysis of *miR-183 *expression. Clinical and pathological data relating to the clinical samples are shown in table 1.

**Table 1:** Breast tumor clinicopathological characteristics

Breast Cancer Clinicopathological Characteristics	Number of Patients N = 70
Median Patient Age yrs (IQR)	52 (48-64)
Median Tumor Size mm (IQR)	25 (20-35)
**Histologic Subtype**	
Invasive Ductal	59
Invasive Lobular	6
Colloid/Mucinous	2
Tubular	2
Papillary	1
**Intrinsic Subtype**	
Luminal A (ER/PR+, HER2/*neu*-)	38
Luminal B (ER/PR+, HER2/*neu*+)	7
HER2 Overexpressing (ER-, PR-, HER2/*neu*+)	10
Triple-Negative (ER-,PR-,HER2/*neu*-)	9
Missing data	6
**Grade**	
1	13
2	17
3	38
Missing data	2
**Nodal Status**	
Node Negative	46
N1	8
N2	11
N3	5
**ER Status**	
-ER Positive	41
-ER Negative	29
**PR Status**	
PR Positive	41
PR Negative	27
Missing data	2
**HER2/*neu *Status**	
HER2 Positive	18
HER2 Negative	47
Missing data	5
**UICC Stage**	
Stage 1	19
Stage 2a	28
Stage 2b	4
Stage 3a	9
Stage 3b	3
Stage 3c	5
Stage 4	2

### Cell Lines and Culture Conditions

The cell lines used in this study included MDA-MB-231 estrogen, progesterone and HER2*/neu *receptor negative (ER-, PR-, HER2*/neu*-), T47D (ER+, PR+, HER2/neu-), SKBR-3 (ER-, PR-, HER2/neu+) and ZR-75-1 (ER+, PR+, HER2*/neu*+). The media used for each cell line were as follows: MDA-MB-231-Lebowitz L15, T47D and ZR-75-1-RPMI-1640, SKBR-3-McCoy-5A. Media was supplemented with 10% FBS and 2 units/mL penicillin G/100 mg/mL streptomycin sulfate. Cells were maintained at 37°C, 5% CO_2 _with a media change twice weekly and passage every 7 days. Prior to transfection experiments, cells were cultured for at least 48 hours in the appropriate antibiotic-free media supplemented with 10% FBS and 1% L-Glutamine.

### Separate miRNA and Large RNA extraction

Large (> 200 nucleotide) and small RNA (< 200 nucleotide) fractions were isolated separately from cultured cells using the RNeasy Plus Mini Kit and RNeasy MinElute^® ^Cleanup Kit (Qiagen, West Sussex, UK) according to the Supplementary Protocol: Purification of miRNA from animal cells. The concentration and purity of RNA were assessed using a NanoDrop™ 1000 spectrophotometer (Nanodrop Technologies, DE, USA). Large RNA integrity was assessed using the RNA 6000 Nano LabChip Series II Assay with the 2100 Bioanalyzer System (Agilent Technologies, Palo Alto, CA, USA) which generated the RNA integrity number (RIN) to ensure that only RNA of good integrity was used in these experiments (RIN range 7.0 - 9.5). Small miRNA enriched fractions were analysed using the Small RNA Assay on the Agilent 2100 Bioanalyzer.

### Quantitative Reverse Transcription of mature miRNAs

RQ-PCR quantification of miRNA expression was performed using TaqMan^® ^MicroRNA Assays according to the manufacturer's protocol (Applied Biosystems, Foster City, CA USA). Small RNA (5 ng) was reverse-transcribed using the MultiScribe™-based High-Capacity cDNA Archive kit (Applied Biosystems). RT negative controls were included in each batch of reactions. PCR reactions were carried out in final volumes of 20μL using an ABI Prism 7900 Sequence Detection System (Applied Biosystems). Briefly, reactions consisted of 1.33μL cDNA, 1X TaqMan^® ^Universal PCR Master Mix, 0.2μM TaqMan^® ^primer-probe mix (Applied Biosystems). Reactions were initiated with a 10 minute incubation at 95°C followed by 40 cycles of 95°C for 15 seconds and 60°C for 60 seconds. *MiRNA-16 *and *let-7a *were used as endogenous controls to standardize miRNA expression[15]. An interassay control derived from a breast cancer cell line (ZR-75-1) was included on each plate. All reactions were performed in triplicate. The threshold standard deviation for intra- and inter-assay replicates was 0.3. Percent PCR amplification efficiencies (E) for each assay were calculated as E = (10-1/slope - 1) × 100, using the slope of the semi-log regression plot of Ct versus log input of cDNA (10-fold dilution series of five points) A threshold of 10% above or below 100% efficiency was applied.

### Quantitative reverse transcription PCR of mRNA

Aliquots of large RNA equivalent to 1μg were reverse transcribed using SuperScript™ III reverse transcriptase (200U/μl, Invitrogen, Calsbad, CA, USA) and random nanomer primers. RQ-PCR reactions were carried out using TaqMan^® ^Gene Expression Assay Primers and an ABI Prism 7900 Sequence Detection System (Applied Biosystems). The transcription levels of *MRPL19 *and *PPIA *were used as endogenous controls[16].

### Relative quantification

The relative quantity of miRNA and mRNA expression was calculated using the comparative Ct (ΔΔCt) method[17]. The geometric mean of the Ct value of the endogenous control genes was used to normalize the data and the lowest expressed sample was used as a calibrator.

### Precursor microRNA Transfection

Hsa-miR-183 pre-miR miRNA precursor (Ambion cat. no. AM17100) was obtained from Ambion. Precursor molecules were transfected into breast cancer cell lines using siPORT Amine transfection reagent (Ambion) and the reverse transfection protocol according to manufacturer's instructions. Culture medium was changed after 24 h of transfection. Transfection effects were evaluated after 72 hrs. Treatment was performed in triplicate. Cells transfected with the Pre-miR™ miRNA precursor molecule - negative control (Ambion, cat. no. AM17110) were used as a control in all transfection/gain-of-function experiments. The efficiency of overexpression of *miR-183 *was evaluated by RQ-PCR.

### Cell proliferation & apoptosis assay

The ApoGlow adenylate nucleotide ratio Assay Kit (Cambrex Bioscience, Nottingham, UK) was used to discriminate between inhibition of proliferation, induction of apoptosis and cell lysis by determination of ADP: ATP ratios, using the luciferin-luciferase bioluminescence assay and in accordance with the manufacturer's instructions. In brief, cells were transfected with hsa-pre-miR-183 or pre-miR-negative control as described, seeded into 96 well plates at a density of 8 × 10^3 ^cells per well and incubated for 72 hours. 100 μL of nucleotide releasing reagent per well was added and the incubation continued for 5 min. Bioluminescence was measured (reading A) with a Luminoskan Ascent^® ^microplate luminometer (Thermo Fisher Scientific) immediately after the addition of 20 μL nucleotide monitoring reagent per well. After 10 min, 20 μL of ADP converting reagent was added per well and luminescence was recorded (reading B). Reading C was obtained 5 min later and ATP: ADP ratios were calculated as A: (C-B). These experiments were performed in triplicate.

### Migration Analysis: In-Vitro Tracking of Cell Migration

For migration assays, cells were used 72 hours after transfection with pre-miR-183 or nonsense pre-miR in basal media. Migration of breast cancer epithelial cells was analysed using Transwell Permeable Supports. A suspension of transfected cells was inoculated into the insert and their migration towards FBS enriched media was quantified. After an18 hour migration incubation, cells remaining above the membrane were removed, the membrane insert was then transferred to ice-cold methanol to fix cells, followed by staining in hematoxylin. Once dry, the membranes were excised from the insert and mounted on slides for quantification of migrated cells (five fields of view on each membrane). Cell counts were done on an Olympus BX60 microscope using analySIS software. Each experiment was repeated, at least in triplicate, with results expressed as mean ± SD.

### Taqman Low Density Array (TLDA)

A TLDA was custom designed and ordered from Applied Biosystems, containing Gene Expression Assays capable of measuring mRNA for 94 breast cancer related genes (96 TaqMan^® ^Gene Expression assay preconfigured in a 384-well format, Part no. 4342261, Lot No A3400, microfluidic cards; Applied Biosystems, Foster City, CA 94404). Each TLDA card was customized with four identical 96 gene sets to facilitate the analysis of two samples per card with 2 replicates per gene and per sample). Each set of 96 genes contained two endogenous controls, *MRPL19 *and *PPIA *(Additional File 1). The TLDA cards were used in a two-step reverse transcriptase polymerase chain reaction (RT-PCR) process using the ABI Prism 7900HT Sequence Detection System (Applied Biosystems) with a TaqMan Low-Density Array Upgrade (Applied Biosystems). Two samples were run on each TLDA card; RNA extracted from cells that were hsa-pre-miR-183 transfected and cells that were pre-miR-negative control transfected. Each cDNA sample at a concentration of 1000 ng in 50 μl was added to an equal volume of TaqMan Universal PCR Master Mix (Applied Biosystems). After gentle mixing, the solution (100μl) was transferred into a loading port on a TLDA card (Applied Biosystems). The loaded TLDA plate was centrifuged and sealed. The Applied Biosystems 7900HT Real-Time PCR system was used to perform the real-time reaction. Thermal cycler conditions were as follows: 2 min at 50°C, 10 min at 94.5°C and 30 s at 97°C, and 1 min at 59.7°C for 40 cycles. The comparative Ct method of relative quantification using MRPL19 and PPIA as endogenous controls was used and relative quantification between hsa-pre-miR-183 transfected samples and negative controls was determined according to the 2^−ΔΔ*C*^_T _method.

### Protein Analysis - Immunocytochemistry

Breast cancer cell lines were transfected with hsa-pre-miR-183 or negative control as described and seeded into chamber slides. After 72 hours the cells were fixed by incubation in ice-cold methanol and immunocytochemical analysis performed using a Ventana Discovery™ automated staining platform with a mouse monoclonal antibody targeting VIL2 (Ezrin) (ab4069, Abcam)

Once staining was completed sections were washed in warm soapy water, dehydrated in serial alcohol immersions and mounted using DPX mounting medium. Changes in VIL2 expression following transfection of breast cancer cells were observed in 5 fields of view using an Olympus BX60 microscope and image analySIS^® ^software.

### Statistical Analysis

All RQ-PCR data were transferred to a statistics computer package (SPSS 14.0 for Windows) for analysis, and the Kolmogorov-Smirnov normality test was applied. Data that were not normally distributed required log transformation to produce an adequate approximation to a normal distribution prior to parametric analysis Comparison of miRNA and mRNA levels between disease groups and transfection conditions was performed using one way ANOVA and independent T-tests. Correlation analysis used Pearson's correlation coefficient and Spearman's Rho for parametric and nonparametric data respectively. Univariate analysis and paired T-tests were used to assess related samples.

## Results

### MiR-183 expression in breast tumors

**Figure 1:**
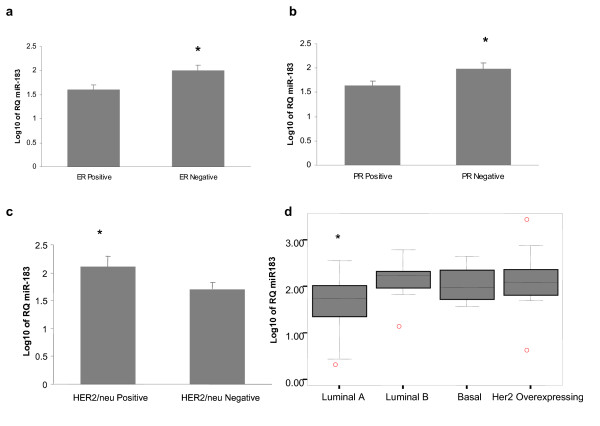
**MiR-183 Expression in Primary Breast Tumors**. **Figure 1a**: *miR-183 *expression is significantly higher in ER-negative tumors *p = 0.01, independent t-test. **Figure 1b**: *miR-183 *expression is signifcantly higher in PR-negative tumors *p = 0.03, independent t-test. **Figure 1c**: *miR-183 *expression is significantly higher in HER2*/neu *positive tumors *p = 0.029, independent t-test. **Figure 1d**: *miR-183 *expression is significantly lower in Luminal A tumors compared to all other subtypes p = 0.02, ANOVA.

The expression of *miR-183 *was quantitated in 70 breast tumors, 9 of which had matched TAN tissue available. RQ-PCR of mature *miR-183 *in these samples showed no significant difference in expression between tumor and tumor associated normal tissue (p = 0.35, paired t-test). Mature *miR-183 *was expressed in all the tumor samples and the expression of *miR-183 *was significantly lower in ER positive breast tumors compared to ER negative tumors with a fold change of 2.97 between the groups, p = 0.01, figure 1a. (mean RQ 87.73 +/-16 vs 260.58+/091 p = 0.01, figure 1a). Conversely, *miR-183 *expression was higher in HER2*/neu *receptor positive tumors compared to HER2*/neu *receptor negative tumors (mean RQ 329.79+/-150 vs 93.33+/-15 p = 0.012, figure 1c). The expression of *miR-183 *is significantly lower in Luminal A tumors (mean RQ 76.26+/-13) than the other intrinsic subtypes, (p = 0.02, ANOVA, figure 1d).

There was a positive correlation between *miR-183 *expression and tumor size but this did not reach statistical significance(R = 0.271, p = 0.076). There was no association of *miR-183 *with other clinicopathological parameters, including histological subtype (p = 0.669), nodal status (p = 0.271), tumor grade (p = 0.326).

### MiR-183 expression in breast cancer cell lines

**Figure 2:**
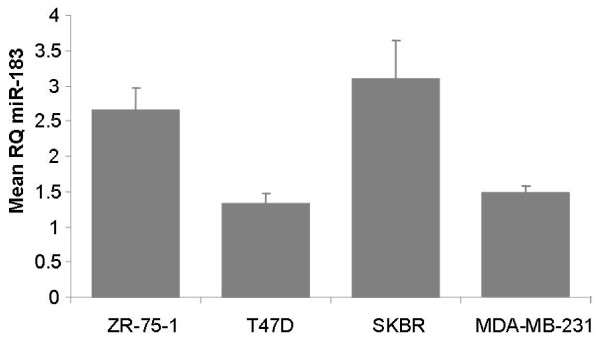
**miR-183 expression in breast cancer cell lines**. The lowest expression of *miR-183 *is in T47D cells which are ER positive and HER2/neu receptor negative

MiRNA was extracted from cell line pellets using the separate purification protocol. The cell lines were chosen on the basis of their ER, PR and HER2*/neu *receptor status and the expression of *miR-183 *in breast cancer cell lines mirrored that of the clinical breast tumor samples, with the lowest expression in T47D cells which are ER+ve/PR+ve and HER2/*neu *-ve, and the highest expression in SKBR-3 cells which are ER-ve/PR-ve and HER2/*neu *+ve (figure 2). This finding suggests that these cell line models of breast cancer subtypes closely reflect tumor miRNA expression patterns and are a useful *in-vitro *breast cancer model for functional analysis of miRNA.

As *miR-183 *was dysregulated in breast tumors in association with clinico-pathological parameters ER, PR and HER2*/neu *status, we further investigated the role of *miR-183 *in breast cancer via gain of function experiments.

**Figure 3:**
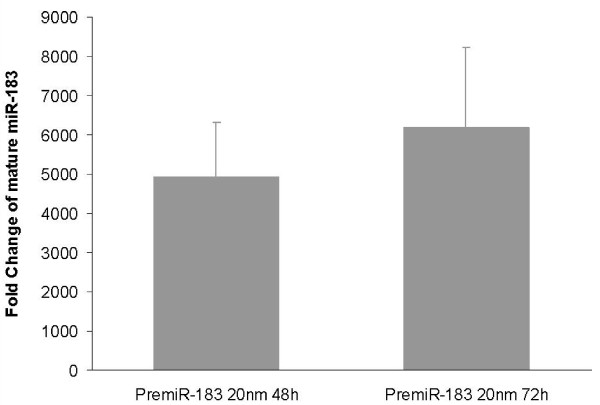
**premiR-183 transfection efficacy**. Mature *miR-183 *levels were quantitated in T47D cells following transfection with PremiR-183 precursor molecules. The fold change represents the level of mature miRNA in PremiR-183 transfected cells relative to cells transfected with PremiR-negative control. Fold change was quantitated using the comparative CT method with *miR-16 *and *let-7a *as endogenous controls and miRNA levels from cells transfected with premiR-negative control as a calibrator.

The efficiency of pre-miR-183 transfection was confirmed by quantitation of mature *miR-183 *levels in miRNA extracted from cells transfected with 20 nm pre-miR-183 and incubated to 48 and 72 hours. The highest mature *miR-183 *levels were seen at 72-hours, with a mean fold change of more than 6000-times that of the negative control (figure 3).

### MiR-183 represses breast cancer cell migration in-vitro

**Figure 4:**
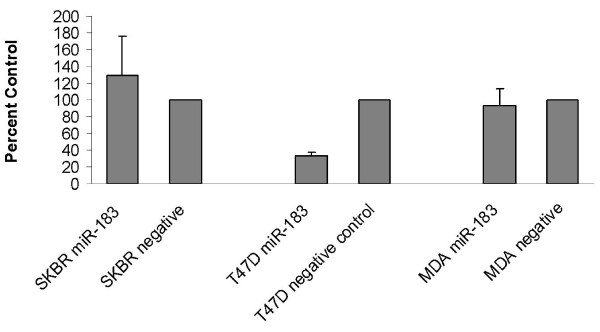
**Overexpression of *miR-183 *inhibits migration in T47D cells**. Quantification of migration through 8- m pore inserts by miR-183 transfected cells as a percentage of that achieved by control cells. The migratory cell number was significantly less in cells transfected with pre-miR-183 compared to cells transfected with pre-miR-negative control (*p = 0.001). In the more aggressive SKBR-3 and MDA-MB-231 cells the migratory cell number was higher overall but there was no difference in migratory number between cells transfected with pre-miR-183 and negative controls. Experiments were performed in triplicate.

To further assess the functional role of *miR-183 *in breast cancer, the effect of *miR-183 *overexpression on cell migration, proliferation and apoptosis were evaluated. Migration of pre-miR-183 and pre-miR-negative control transfected cells in response to FBS enriched media was tracked using Transwell inserts. T47D cells transfected with pre-miR-183 showed a significant decrease in migration compared to negative controls. Cell migration was quantified in five fields of view on each membrane (figure 4).

### MiR-183 does not affect proliferation or apoptosis in breast cancer cells

The ApoGlow™ Adenylate Nucleotide Ratio Assay (Cambrex) was used to determine the effect of *miR-183 *overexpression on distinct mechanisms underlying alterations in cell viability. This luciferase-based assay allows the efficient detection, quantitation and distinction between apoptosis, necrosis, growth-arrest and cell proliferation. ATP is a biomarker for the presence of metabolically active cells, indicative of cell health and viability and is rapidly consumed if cells are compromised. There was no significant difference in ATP or ADP levels between *miR-183 *transfected cells and controls at 72 hours indicating no effect of miR-183 on cell proliferation, apoptosis or viability.

### Changes in gene expression in the presence of increased miR-183

**Figure 5:**
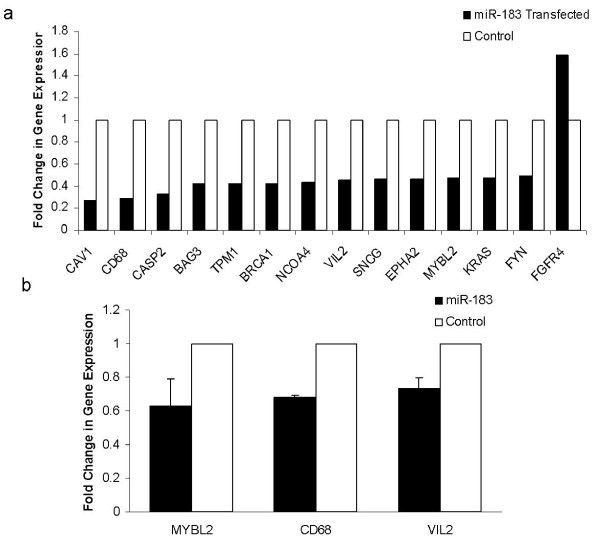
**Dysregulation of mRNAs following induced overexpression of *miR-183***. **Figure 5a**: 14 genes were differentially expressed in T47D cells transfected with pre-*miR-183 *compared to negative control. Gene expression was analysed using TLDA. **Figure 5b**: RQ-PCR using conventional taqman assays verified downregulation of a selection of breast cancer associated genes following transfection of T47D cells with pre-miR-183.

To further explore the role of *miR-183 *in breast cancer, we compared the expression levels of 94 breast cancer related genes in cells transfected with pre-miR-183 and pre-miR-negative control. T47D cells were used in this experiment as they express he lowest levels of endogenous *miR-183*. Cells were reverse transfected, incubated for 72 hours and RNA extracted as described. Gene expression was quantitated by RQ-PCR on a customized Taqman Low Density Array as described. The fold change in gene expression in cells overexpressing *miR-183 *was calculated relative to the negative control cells using the comparative Ct method (figure 5a) and the percentage dysregulation was calculated using the equation : % downregulation = 100-100 × 2^-ΔΔCt^. With the criterion of dysregulation of > 50%, we identified 14 genes that were differentially expressed following transfection of pre-miR-183 compared to negative controls. Among these genes, there were genes functioning in cell growth, migration, proliferation and apoptosis, functions which are inextricably linked to tumor progression (table 2). The dysregulation of a selection of these genes was validated by repeating the transfection in triplicate and quantitating gene expression using conventional individual Taqman assays. RQ-PCR verified that the mRNA level of VIl2, MYBL2 and CD68 could be downregulated by overexpression of *miR-183 *(figure 5b).

**Table 2:** Dysregulated genes following overexpression of *miR-183*

Gene	Dysregulation (%)	Function	Putative Target
CAV1	73.12	Tumor suppressor gene candidate. Codes for scaffolding protein	No miRBase No Pictar NoTargetscan
CD68	70.51	Phagocytosis	No miRBase No Pictar NoTargetscan
CASP2	67.16	Apoptosis	Yes miRBase No Pictar Yes Targetscan
BAG3	57.70	Negative regulator of apoptosis, adhesion, migration	Yes miRBase No Pictar No Targetscan
TPM1	57.53	Actin-binding. Stabilizes cytoskeleton actin filaments	No miRBase No Pictar Yes Targetscan
BRCA1	57.21	Tumor suppressor, DNA repair	No miRBase No Pictar No Targetscan
NCOA4	56.08	Ligand-independent coactivator of PPAR-gamma	No miRBase No Pictar Yes Targetscan
**VIL2	54.51	Linker between plasma membrane and actin cytoskeleton, involved in cell adhesion & migration.	Yes miRBase Yes Pictar Yes targetscan
SNCG	53.69	Role in neurofilament network integrity.	No miRBase No Pictar No Targetscan
EPHA2	53.15	Receptor for members of the ephrin-A family	No miRBase No Pictar Yes Targetscan
MYBL2	52.82	Transcription factor involved in the regulation of cell survival, proliferation, and differentiation	No miRBase No Pictar No Targetscan
KRAS	52.30	Ras proteins bind GDP/GTP and possess intrinsic GTPase activity	No miRBase No Pictar No Targetscan
FYN	50.91	Implicated in the control of cell growth	No miRBase No Pictar No Targetscan
FGFR4	-58.97	Implicated in cell growth, proliferation.	No miRBase No Pictar No Targetscan

These genes could be either directly or indirectly affected by *miR-183*. To further investigate this, we employed *in silico *analysis using three target prediction programmes (miRBase, TargetScan and PicTar)[18-20]. VIL2 was the only gene that was predicted to have a binding site for *miR-183 *in its 3'UTR by all three databases, indicating that VIL2 is likely to be directly affected by *miR-183*.

### Overexpression of miR-183 alters VIL2 protein expression in breast cancer cells

To further investigate the nature of VIL2 as a potential *miR-183 *target, we evaluated the effect of increased *miR-183 *on VIL2 protein expression in T47D and SKBR3 cells which have distinct hormone and HER2*/neu *receptor status profiles. Cells were transfected with pre-miR-183 or pre-miR negative control, seeded onto chamber slides which were then fixed and stained with VIL2 antibody at 72 hours. The proportion and intensity of staining was evaluated in 5 fields of view using an Olympus BX60 microscope.

**Figure 6:**
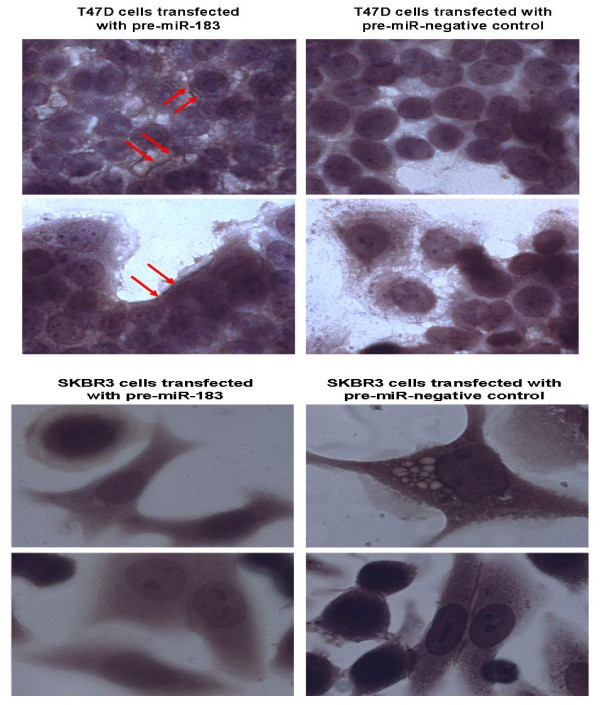
**VIL2 expression in breast cancer cells overexpressing miR-183**. **Figure 6a**: stronger membranous staining of VIL2 in cells transfected with premiR-183 is denoted by the red arrows. **Figure 6b**: VIL2 staining is weaker in cells transfected with premiR-183.

There was positive staining of both pre-miR-183 transfected and negative control T47D cells however membranous staining of VIL2 appeared stronger in T47D cells transfected with pre-miR-183 (figure 6a).

In SKBR3 cells, there was reduced cytoplasmic and membranous staining of VIL2 in cells transfected with pre-miR-183 compared to negative controls (figure 6b). This indicates that *miR-183 *affects VIL2 at both the mRNA and protein levels.

## Discussion

MiRNA expression patterns are altered in breast cancer as previously described[13,21-24], and expression profiling of miRNA to classify tumors according to clinicopathological variables appears to have superior accuracy to mRNA profiling[8]. This evidence underlines the role of miRNAs in breast cancer molecular biology and provides the starting point for exploration of novel molecular mechanisms.

Quantitation of *miR-183 *by RQ-PCR in primary breast tumors revealed that *miR-183 *expression is significantly lower in ER and PR positive compared to ER and PR negative breast tumors, while *miR-183 *expression was significantly higher in HER2/*neu *positive compared to HER2/*neu *negative tumors. Luminal A tumors exhibited the lowest expression of *miR-183*. This is the first report of *miR-183 *dysregulation in association with hormone and HER2/*neu *receptor status in clinical breast tumour samples. Differential expression of *miR-183 *in breast cancer cell lines has been demonstrated by Sempere *et al *[25] using northern blotting, with decreased expression in basal-type compared to luminal-type cell lines. Interestingly, the expression of *miR-182 *which is co-ordinately expressed from the same genetic locus as *miR-183 *has been shown to be downregulated in ER positive tumors[13], this supports our finding of *miR-183 *dysregulation in association with ER status. The identification of dysregulated miRNAs in relation to ER and HER2/neu receptor status in breast cancer is of great clinical significance as the specific combination of receptor status has implications for the current classification and targeted therapy of breast cancer. The past decade has seen the development of a molecular taxonomy of breast cancer which has been divided into subtypes based on the expression of specific genes, including ER and HER2/neu. However, this taxonomy remains limited because clinical outcome may be variable within each subtype, suggesting the existence of further divisions that are yet to be characterised; for example, subgroups have been identified within the Luminal A and ER positive breast tumours which have distinct survival and prognostic profiles[26,27]. One obvious explanation for the lack of homogeneity within the breast cancer subtypes is the potential dysregulation of miRNAs between and within subgroups. MiRNAs represent a new layer of molecular complexity in breast cancer and miRNA expression profiles are thought to be more accurate than mRNA at classifying tumours[8]. Their unique ability to requlate gene expression confers functionality on these molecules, and when dysregulated in breast cancer they may be responsible for altering the phenotype of breast tumours. Thus, it is important to functionally characterise miRNAs, such as *miR-183 *that are dysregulated in breast cancer and establish what effect this dysregulation may have on tumour behaviour.

MiR-183 is located on chromosome 7q32.3 and is part of a miRNA family comprised of three homologous miRNAs (*miR-183, miR-182 *and *miR-96*) that are co-ordinately expressed from this locus. *MiR-183 *family members have been shown to be upregulated in leukaemia, hepatic and colorectal cancer [9-12]. Conversely, *miR-183 *has been shown to be downregulated and inversely associated with metastasis in lung cancer[14]. This evidence indicates that the expression patterns of *miR-183 *are tissue specific, and that this miRNA may have divergent functions depending on the tissue or cell type. Such context dependence of miRNA function has previously been reported in the results of biochemical studies indicating that miRNA repression of mRNA is dependent on the specific cellular conditions[28].

There has been limited functional analysis of *miR-183*; it has been postulated that *miR-183 *may downregulate anti-apoptotic factors including forkhead transcription factor (FOX) proteins, suggesting a pro-apoptotic function for this miRNA[9,29], however this was based on computational analysis and no experimental verification was provided.

During the course of these experiments, the first functional study of *miR-183 *in malignancy was reported; Wang *et al*[14] identified *miR-183 *as a potential metastasis inhibitor in lung cancer and demonstrated that over expression of *miR-183 *inhibited migration of lung cancer cells. They reported *miR-183 *induced dysregulation of expression of genes involved in migration and invasion, including VIL2. Overexpression of *miR-183 *in HeLa cells has also been shown to inhibit migration and invasion, however this was shown to be mediated through direct targeting of integrin β1 (ITGB1)[30], indicating that *miR-183 *is likely to have a number of mRNA targets through which it mediates biological effects in cancer cells.

The functional role of *miR-183 *in breast cancer was investigated in these experiments using a gain-of-function approach. The lowest endogenous expression of *miR-183 *was observed in T47D cells, which are ER and PR positive and HER2/*neu *negative, making these cells a suitable *in-vitro *representation of the Luminal A subtype of breast cancer.

The effect of *miR-183 *modulation on cellular functions which are frequently dysregulated in malignancy was assessed in T47D cells (ER^+^, PR^+^, HER2*/neu*^-^), SKBR3 cells (ER^-^, PR^-^, HER2*/neu*^+^), and MDA-MB-231(ER^-^, PR^-^, HER2*/neu*^-^), cells which exhibit distinct hormone and HER2*/neu *receptor profiles. There was no effect on apoptosis or proliferation, which refutes the hypothesis of *miR-183 *having a pro-apoptotic function. Induced overexpression of *miR-183 *resulted in decreased migration of T47D cells in transwell assays, but there was no significant change in migration of SKBR3 or MDA-MB-231 cells (figure 4).

The difference in effect noted between the cell lines is not completely surprising for a number of reasons; firstly T47D cells were shown to express the lowest endogenous levels of *miR-183*, thus the effect of overexpression in these cells would be expected to result in a more dramatic or measurable phenotypic effect than in cells where *miR-183 *is expressed at higher levels endogenously. Furthermore, although the three cell lines are epithelial breast cancer cell lines, they are unrelated and are phenotypically distinct; differences in cell behaviour would thus be expected that relate to the previously mentioned cell-type specific regulatory mechanisms and actions of miRNA. As *miR-183 *was shown to be dysregulated in relation to ER and HER2/*neu *status it is not unexpected that this miRNA would behave differently depending on the cell type and environment, particularly in relation to the expression of these receptors.

The finding that *miR-183 *reduces migration in breast cancer cells indicates that this miRNA may play an important role in the suppression of metastases, as cell motility and migration are key steps in the metastatic cascade. These findings are in concordance with the findings of Wang *et al*[14] who demonstrated a similar effect in lung cancer cells. Increasing evidence supports a role for multiple miRNAs in the regulation of metastases in breast cancer[31-35]. This is hugely significant as metastasis is the overwhelming cause of mortality in breast cancer patients, but a complete understanding of its molecular and cellular determinants remains elusive[36]. Gene expression profiling studies have provided gene-sets that are predictive of breast cancer progression and prognosis[37], however the regulatory networks that establish such altered gene expression states are only now coming into focus. It is important that the targets and regulators of metastasis involved miRNAs are elucidated to improve our understanding of the specific roles played by miRNAs in the complex, multistep process of primary tumor invasion and metastasis.

To further elucidate the mechanism by which *miR-183 *regulates metastasis we analysed the expression of 96 breast cancer related genes using a low density array and identified candidate genes which were dysregulated following transfection of T47D cells with *miR-183 *(table 2). T47D cells were chosen for this part of the experiment as they had shown the greatest phenotypic change in response to *miR-183 *modulation. The finding of genes that are both downregulated and upregulated is in keeping with recent reports that miRNAs are differential modulators of gene expression and can activate mRNA translation in addition repression [38]. Functions of the changed genes were analysed using NetAffx Gene Ontology Mining Tool available at the Affymetrix website http://www.affymetrix.com/ and a number of the genes were found to have roles in migration and maintenance of the actin cytoskeleton integrity. These data support the hypothesis that regulation of metastasis requires coordinated expression of multiple genes. Computational prediction algorithms were used to identify which of these miRNAs may be direct targets of *miR-183 *via complementary binding at the 3'UTR. All three of the algorithms used (miRBase, PicTar and TargetScan) predicted VIL2/Ezrin a member of the ezrin/radixin/moesin (ERM) family of proteins that mediate cytoskeletal-membrane interactions and cell signalling[39] to be a target of *miR-183 *via perfect complementarity at a binding site at position 366-373 of Ezrin 3'UTR. Downregulation of VIL2 as a result of *miR-183 *overexpression was validated using RQ-PCR, indicating that regulation occurred at the mRNA level, this is in contrast to the findings of Wang *et al*[14] who found regulation of Ezrin to be at the posttranscriptional level in lung cancer. We also found changes in Ezrin protein expression in breast cancer cells following transfection with *miR-183*.

It is likely that much of the migration inhibition induced by *miR-183 *in breast cancer cells is modulated via Ezrin downregulation. Ezrin is a membrane cytoskeleton crosslinker which plays a role in cell functions including control of the actin cytoskeleton, adhesion and motility. The actin cytoskeleton is a highly dynamic network of actin polymers and proteins and is required for cell motility, cortical organisation and cell division. Ezrin has been shown to be a key component in tumor metastasis [40,41] and is associated with metastatic potential and poor outcome in breast carcinomas[42,43].

The assessment of Ezrin expression in breast cancer cells can be both cytoplasmic and membranous, however the differential localization, rather than total Ezrin protein levels is associated with the malignant potential of the cell. In an immunohistochemical analysis of Ezrin in primary breast tumors and lymph node metastases, expression levels of Ezrin protein were significantly higher in primary cancer tissues than lymph node metastases but the Ezrin staining pattern was seen mainly in the membrane region of primary cancer cells, whereas mainly in cytosols of metastatic cancer cells, indicating that the subcellular location of Ezrin protein in breast cancer is integral to the metastatic potential of the cell [44]. Interestingly, the immunocytochemical analysis of T47D cells for Ezrin expression following transfection with *miR-183 *demonstrated more membranous staining in the transfected cells, which is in keeping with less metastatic potential (figure 6a). These were the cells that demonstrated decreased migration on functional analysis. In the SKBR-3 cells which did not exhibit decreased migration, there appeared to be decreased membranous and cytoplasmic expression of Ezrin in the transfected cells (figure 6b), this suggests that the overexpression of *miR-183 *in these cells had disrupted Ezrin expression but this had not translated to a measurable functional effect.

These results imply that there is an important regulatory role of *miR-183 *in breast cancer, centering on cell migration and metastatic potential.

## Conclusion

The identification of the molecular mechanisms responsible for aggressive tumor behaviour and metastasis is important for understanding disease progression, guiding disease management, and developing potentially novel cancer treatment strategies. The elucidation of the functional role of *miR-183 *in breast cancer promises to provide an important avenue for further analysis in in-vivo systems with an aim to develop a new diagnostic and therapeutic target in the management of metastatic breast cancer.

## List of Abbreviations

**ADP**: Adenosine diphosphate; **ATP**: Adenosine triphosphate; **cDNA**: Complementary DNA; **Ch7q32**: Chromosomal location 7q32; **Ct**: Cycle threshold; **ΔCt**: Comparative Cycle threshold; **°C**: Degrees Celsius; **E**: PCR Amplification Efficiencies; **ER**: Estrogen Receptor; **FBS**: Fetal Bovine Serum; **HER2/neu**: v-erb-b2 erythroblastic leukemia viral oncogene homolog 2 receptors; **miRNA**: microRNA; **mRNA**: Messenger RNA; **mg**: Milligrams; **MYBL2**: v-myb avian myeloblastosis viral oncogene homolog-like 2; **mL**: Millilitres; **Ng**: Nanograms; **μg**: micrograms; **μL**: microlitres; **PR**: Progesterone Receptor; **RQ-PCR**: Real Time Quantitative Polymerase Chain Reaction; **RNA**: Ribonucleic Acid; **RIN**: RNA Integrity Number; **RT**: Reverse Transcription; **TLDA**: Taqman Low Density Array; **VIL2**: Villin 2

## Competing interests

The authors declare that they have no competing interests.

## Authors' contributions

AJL performed the experiments, was responsible for data analyses and drafted the manuscript. NM conceived, designed and supervised experimental work and manuscript editing. RMD designed and supervised experimental work. MJK contributed throughout the experiment and critically reviewed the manuscript. All authors read and approved the final manuscript.

## Pre-publication history

The pre-publication history for this paper can be accessed here:

http://www.biomedcentral.com/1471-2407/10/502/prepub

## Supplementary Material

Additional File 1 **96 Genes on Customized Low Density Array (TLDA) Card**. Details of the 96 breast and cancer associated genes (including two endogenous controls) included on the customized TLDA microfluicidic card which were quantitated in cells transfected with premiR-183 and negative controls. Click here for file
