# Supplementary material for: Dysregulated miR-183 inhibits migration in breast cancer cells
Source: BMC Cancer. 2010 Sep 21;10:502. doi: 10.1186/1471-2407-10-502 (PMC2955037; doi:10.1186/1471-2407-10-502)
Supplement: Additional File 1 — 96 Genes on Customized Low Density Array (TLDA) Card. Details of the 96 breast and cancer associated genes (including two endogenous controls) included on the customized TLDA microfluicidic card which were quantitated in cells transfected with premiR-183 and negative controls. [file 1471-2407-10-502-S1.DOC]

**Supplementary Material**

Genes quantitated in miR-183 transfected cells using Customized Taqman Low Density Array

| **GENE** | **Description** | **Detector** |
| --- | --- | --- |
| ACVR1 | activin A receptor type 1precursor | Hs00153836_m1 |
| AHRR | aryl-hydrocarbon receptor repressor, Programmed cell death protein 6 | Hs00324967_m1 |
| AKT1 | RAC-alpha serine/threonine-protein kinase | [Hs00178289_m1](https://products.appliedbiosystems.com/ab/en/US/adirect/ab?cmd=ABAssayDetailDisplay&assayID=Hs00178289_m1&Fs=y&adv_phrase3=EXACT&adv_phrase2=EXACT&adv_phrase1=EXACT&assayType=ge&catID=601267&SearchRequest.Common.SortSpec=&searchValue=null&MFCSpeciesType=null&searchBy=null&adv_kw_filter3=GENE_SYMBOL&srchType=keyword&adv_kw_filter2=GENE_SYMBOL&group1=noValue&SearchRequest.Common.QueryText=AKT1&adv_kw_filter1=GENE_SYMBOL&inventoried=*&adv_query_text3=&species=Homo+sapiens&adv_query_text2=&adv_query_text1=&paraTreeViewNode=null&adv_boolean3=AND&displayAdvSearchResults=&SearchRequest.Common.ResultsPerPage=25&adv_boolean2=AND&adv_boolean1=AND&chkBatchQueryText=false&kwfilter=GENE_SYMBOL&SearchRequest.Common.PageNumber=1&formatType=default&isSL=N& ) |
| AXL | Tyrosine-protein kinase receptor UFO precursor, oncogene | Hs00242357_m1 |
| BAG1 | BCL2-associated athanogene | Hs00185390_m1 |
| BAG3 | BCL2-associated athanogene 3 | Hs00188713_m1 |
| BCL2 | B-Cell CLL/Lymphoma 2 | Hs00153350_m1 |
| BIN1 | bridging integrator 1 | Hs00184913_m1 |
| BIRC5 | (survivin) Baculoviral IAP Repeat Containing-5 | Hs00153353_m1 |
| BRAF | B-Raf proto-oncogene serine/threonine-protein kinase | [Hs00269944_m1](https://products.appliedbiosystems.com/ab/en/US/adirect/ab?cmd=ABAssayDetailDisplay&assayID=Hs00269944_m1&Fs=y&adv_phrase3=EXACT&adv_phrase2=EXACT&adv_phrase1=EXACT&assayType=ge&catID=601267&SearchRequest.Common.SortSpec=&searchValue=null&MFCSpeciesType=null&searchBy=null&adv_kw_filter3=all&srchType=keyword&adv_kw_filter2=all&group1=noValue&SearchRequest.Common.QueryText=BRAF&adv_kw_filter1=all&inventoried=1&adv_query_text3=&species=Homo+sapiens&adv_query_text2=&adv_query_text1=&paraTreeViewNode=null&adv_boolean3=AND&displayAdvSearchResults=&SearchRequest.Common.ResultsPerPage=25&adv_boolean2=AND&adv_boolean1=AND&chkBatchQueryText=false&kwfilter=all&SearchRequest.Common.PageNumber=1&formatType=default&isSL=N& ) |
| BRAP | BRCA1 associated protein | Hs00198914_m1 |
| BRCA1 | Breast cancer type 1 susceptibility protein | [Hs00173233_m1](https://products.appliedbiosystems.com/ab/en/US/adirect/ab?cmd=ABAssayDetailDisplay&assayID=Hs00173233_m1&Fs=y&adv_phrase3=EXACT&adv_phrase2=EXACT&adv_phrase1=EXACT&assayType=ge&catID=601267&SearchRequest.Common.SortSpec=&searchValue=null&MFCSpeciesType=null&searchBy=null&adv_kw_filter3=GENE_SYMBOL&srchType=keyword&adv_kw_filter2=GENE_SYMBOL&group1=noValue&SearchRequest.Common.QueryText=BRCA1&adv_kw_filter1=GENE_SYMBOL&inventoried=*&adv_query_text3=&species=Homo+sapiens&adv_query_text2=&adv_query_text1=&paraTreeViewNode=null&adv_boolean3=AND&displayAdvSearchResults=&SearchRequest.Common.ResultsPerPage=25&adv_boolean2=AND&adv_boolean1=AND&chkBatchQueryText=false&kwfilter=GENE_SYMBOL&SearchRequest.Common.PageNumber=1&formatType=default&isSL=N& ) |
| BRMS1 | breast cancer metastasis suppressor 1 | Hs00363036_m1 |
| CASP10 | caspase 10, apoptosis-related cysteine peptidase | Hs01017902_m1 |
| CASP2 | caspase 2, apoptosis-related cysteine peptidase | Hs00234982_m1 |
| CAV1 | Caveolin-1. | [Hs00971716_m1](https://products.appliedbiosystems.com/ab/en/US/adirect/ab?cmd=ABAssayDetailDisplay&assayID=Hs00971716_m1&Fs=y&adv_phrase3=EXACT&adv_phrase2=EXACT&adv_phrase1=EXACT&assayType=ge&catID=601267&SearchRequest.Common.SortSpec=&searchValue=null&MFCSpeciesType=null&searchBy=null&adv_kw_filter3=GENE_SYMBOL&srchType=keyword&adv_kw_filter2=GENE_SYMBOL&group1=noValue&SearchRequest.Common.QueryText=CAV1&adv_kw_filter1=GENE_SYMBOL&inventoried=*&adv_query_text3=&species=Homo+sapiens&adv_query_text2=&adv_query_text1=&paraTreeViewNode=null&adv_boolean3=AND&displayAdvSearchResults=&SearchRequest.Common.ResultsPerPage=25&adv_boolean2=AND&adv_boolean1=AND&chkBatchQueryText=false&kwfilter=GENE_SYMBOL&SearchRequest.Common.PageNumber=1&formatType=default&isSL=N& ) |
| CCNB1 | cyclin B1 | Hs00259126_m1 |
| CCND1 | G1/S-specific cyclin-D1 (PRAD1 oncogene) (BCL-1 oncogene). | [Hs00277039_m1](https://products.appliedbiosystems.com/ab/en/US/adirect/ab?cmd=ABAssayDetailDisplay&assayID=Hs00277039_m1&Fs=y&adv_phrase3=EXACT&adv_phrase2=EXACT&adv_phrase1=EXACT&assayType=ge&catID=601267&SearchRequest.Common.SortSpec=&searchValue=null&MFCSpeciesType=null&searchBy=null&adv_kw_filter3=GENE_SYMBOL&srchType=keyword&adv_kw_filter2=GENE_SYMBOL&group1=noValue&SearchRequest.Common.QueryText=CCND1&adv_kw_filter1=GENE_SYMBOL&inventoried=*&adv_query_text3=&species=Homo+sapiens&adv_query_text2=&adv_query_text1=&paraTreeViewNode=null&adv_boolean3=AND&displayAdvSearchResults=&SearchRequest.Common.ResultsPerPage=25&adv_boolean2=AND&adv_boolean1=AND&chkBatchQueryText=false&kwfilter=GENE_SYMBOL&SearchRequest.Common.PageNumber=1&formatType=default&isSL=N& ) |
| CCNE1 | G1/S-specific cyclin-E1 | [Hs01026536_m1](https://products.appliedbiosystems.com/ab/en/US/adirect/ab?cmd=ABAssayDetailDisplay&assayID=Hs01026536_m1&Fs=y&adv_phrase3=EXACT&adv_phrase2=EXACT&adv_phrase1=EXACT&assayType=ge&catID=601267&SearchRequest.Common.SortSpec=&searchValue=null&MFCSpeciesType=null&searchBy=null&adv_kw_filter3=GENE_SYMBOL&srchType=keyword&adv_kw_filter2=GENE_SYMBOL&group1=noValue&SearchRequest.Common.QueryText=CCNE1&adv_kw_filter1=GENE_SYMBOL&inventoried=*&adv_query_text3=&species=Homo+sapiens&adv_query_text2=&adv_query_text1=&paraTreeViewNode=null&adv_boolean3=AND&displayAdvSearchResults=&SearchRequest.Common.ResultsPerPage=25&adv_boolean2=AND&adv_boolean1=AND&chkBatchQueryText=false&kwfilter=GENE_SYMBOL&SearchRequest.Common.PageNumber=1&formatType=default&isSL=N& ) |
| CD40 | CD40 molecule, TNF receptor superfamily member 5 | Hs00374176_m1 |
| CD68 | CD68 | Hs00154355_m1 |
| Chek2 | CHK2 checkpoint homolog (S. pombe) | Hs00418065_m1 |
| CTSL2 | Cathepsin L2 | Hs00426731_m1 |
| CXCL12 | Stromal cell-derived factor 1 precursor (SDF-1) | [Hs00171022_m1](https://products.appliedbiosystems.com/ab/en/US/adirect/ab?cmd=ABAssayDetailDisplay&assayID=Hs00171022_m1&Fs=y&adv_phrase3=EXACT&adv_phrase2=EXACT&adv_phrase1=EXACT&assayType=ge&catID=601267&SearchRequest.Common.SortSpec=&searchValue=null&MFCSpeciesType=null&searchBy=null&adv_kw_filter3=GENE_SYMBOL&srchType=keyword&adv_kw_filter2=GENE_SYMBOL&group1=noValue&SearchRequest.Common.QueryText=CXCL12&adv_kw_filter1=GENE_SYMBOL&inventoried=1&adv_query_text3=&species=Homo+sapiens&adv_query_text2=&adv_query_text1=&paraTreeViewNode=null&adv_boolean3=AND&displayAdvSearchResults=&SearchRequest.Common.ResultsPerPage=25&adv_boolean2=AND&adv_boolean1=AND&chkBatchQueryText=false&kwfilter=GENE_SYMBOL&SearchRequest.Common.PageNumber=1&formatType=default&isSL=N& ) |
| E2F4 | Transcription factor E2F4 (E2F-4). | [Hs00608098_m1](https://products.appliedbiosystems.com/ab/en/US/adirect/ab?cmd=ABAssayDetailDisplay&assayID=Hs00608098_m1&Fs=y&adv_phrase3=EXACT&adv_phrase2=EXACT&adv_phrase1=EXACT&assayType=ge&catID=601267&SearchRequest.Common.SortSpec=&searchValue=null&MFCSpeciesType=null&searchBy=null&adv_kw_filter3=GENE_SYMBOL&srchType=keyword&adv_kw_filter2=GENE_SYMBOL&group1=noValue&SearchRequest.Common.QueryText=E2F4&adv_kw_filter1=GENE_SYMBOL&inventoried=1&adv_query_text3=&species=Homo+sapiens&adv_query_text2=&adv_query_text1=&paraTreeViewNode=null&adv_boolean3=AND&displayAdvSearchResults=&SearchRequest.Common.ResultsPerPage=25&adv_boolean2=AND&adv_boolean1=AND&chkBatchQueryText=false&kwfilter=GENE_SYMBOL&SearchRequest.Common.PageNumber=1&formatType=default&isSL=N& ) |
| EGFR | Epidermal growth factor receptor precursor (Receptor tyrosine-protein kinase ErbB-1). | Hs00193306_m1 |
| ELK1 | ETS domain-containing protein Elk-1 | [Hs00901849_g1](https://products.appliedbiosystems.com/ab/en/US/adirect/ab?cmd=ABAssayDetailDisplay&assayID=Hs00901849_g1&Fs=y&adv_phrase3=EXACT&adv_phrase2=EXACT&adv_phrase1=EXACT&assayType=ge&catID=601267&SearchRequest.Common.SortSpec=&searchValue=null&MFCSpeciesType=null&searchBy=null&adv_kw_filter3=GENE_SYMBOL&srchType=keyword&adv_kw_filter2=GENE_SYMBOL&group1=noValue&SearchRequest.Common.QueryText=ELK1&adv_kw_filter1=GENE_SYMBOL&inventoried=*&adv_query_text3=&species=Homo+sapiens&adv_query_text2=&adv_query_text1=&paraTreeViewNode=null&adv_boolean3=AND&displayAdvSearchResults=&SearchRequest.Common.ResultsPerPage=25&adv_boolean2=AND&adv_boolean1=AND&chkBatchQueryText=false&kwfilter=GENE_SYMBOL&SearchRequest.Common.PageNumber=1&formatType=default&isSL=N& ) |
| EP300 | E1A-associated protein p300). | [Hs00230938_m1](https://products.appliedbiosystems.com/ab/en/US/adirect/ab?cmd=ABAssayDetailDisplay&assayID=Hs00230938_m1&Fs=y&adv_phrase3=EXACT&adv_phrase2=EXACT&adv_phrase1=EXACT&assayType=ge&catID=601267&SearchRequest.Common.SortSpec=&searchValue=null&MFCSpeciesType=null&searchBy=null&adv_kw_filter3=GENE_SYMBOL&srchType=keyword&adv_kw_filter2=GENE_SYMBOL&group1=noValue&SearchRequest.Common.QueryText=EP300&adv_kw_filter1=GENE_SYMBOL&adv_query_text3=&species=Homo+sapiens&adv_query_text2=&adv_query_text1=&paraTreeViewNode=null&adv_boolean3=AND&displayAdvSearchResults=&adv_boolean2=AND&adv_boolean1=AND&chkBatchQueryText=false&kwfilter=GENE_SYMBOL&SearchRequest.Common.PageNumber=1&formatType=default&isSL=N& ) |
| EPHA2 | Ephrin type-A receptor 2 precursor | Hs00171656_m1 |
| EPHB2 | Ephrin type-B receptor 2 precursor | [Hs00362096_m1](https://products.appliedbiosystems.com/ab/en/US/adirect/ab?cmd=ABAssayDetailDisplay&assayID=Hs00362096_m1&Fs=y&adv_phrase3=EXACT&adv_phrase2=EXACT&adv_phrase1=EXACT&assayType=ge&catID=601267&SearchRequest.Common.SortSpec=&searchValue=null&MFCSpeciesType=null&searchBy=null&adv_kw_filter3=all&srchType=keyword&adv_kw_filter2=all&group1=noValue&SearchRequest.Common.QueryText=EPHB2&adv_kw_filter1=all&inventoried=*&adv_query_text3=&species=Homo+sapiens&adv_query_text2=&adv_query_text1=&paraTreeViewNode=null&adv_boolean3=AND&displayAdvSearchResults=&SearchRequest.Common.ResultsPerPage=25&adv_boolean2=AND&adv_boolean1=AND&chkBatchQueryText=false&kwfilter=all&SearchRequest.Common.PageNumber=1&formatType=default&isSL=N& ) |
| ERa (ESR1) | Estrogen Receptor alpha | Hs00174860_m1 |
| ERb (ESR2) | Estrogen Receptor beta | Hs00230957_m1 |
| ERBB2 / HER2 | Human Epidermal Growth Factor 2 | Hs00170433_m1 |
| ERBB3 | v-erb-b2 erythroblastic leukemia viral oncogene homolog 3 (avian) | Hs00176538_m1 |
| ESRRA | Steroid hormone receptor ERR1 (Estrogen-related receptor, alpha) | Hs00607062_gH |
| FGFR4 | Fibroblast growth factor receptor 4 precursor | Hs00608745_m1 |
| FIGF | Vascular endothelial growth factor D precursor (VEGF-D) | Hs00189521_m1 |
| FOS | Proto-oncogene protein c-fos (Cellular oncogene fos) | [Hs00170630_m1](https://products.appliedbiosystems.com/ab/en/US/adirect/ab?cmd=ABAssayDetailDisplay&assayID=Hs00170630_m1&Fs=y&adv_phrase3=EXACT&adv_phrase2=EXACT&adv_phrase1=EXACT&assayType=ge&catID=601267&SearchRequest.Common.SortSpec=&searchValue=null&MFCSpeciesType=null&searchBy=null&adv_kw_filter3=GENE_SYMBOL&srchType=keyword&adv_kw_filter2=GENE_SYMBOL&group1=noValue&SearchRequest.Common.QueryText=FOS&adv_kw_filter1=GENE_SYMBOL&inventoried=*&adv_query_text3=&species=Homo+sapiens&adv_query_text2=&adv_query_text1=&paraTreeViewNode=null&adv_boolean3=AND&displayAdvSearchResults=&SearchRequest.Common.ResultsPerPage=25&adv_boolean2=AND&adv_boolean1=AND&chkBatchQueryText=false&kwfilter=GENE_SYMBOL&SearchRequest.Common.PageNumber=1&formatType=default&isSL=N& ) |
| FOXA1 | Hepatocyte nuclear factor 3-alpha (HNF-3A) (Forkhead box protein A1). | [Hs00270129_m1](https://products.appliedbiosystems.com/ab/en/US/adirect/ab?cmd=ABAssayDetailDisplay&assayID=Hs00270129_m1&Fs=y&adv_phrase3=EXACT&adv_phrase2=EXACT&adv_phrase1=EXACT&assayType=ge&catID=601267&SearchRequest.Common.SortSpec=&searchValue=null&MFCSpeciesType=null&searchBy=null&adv_kw_filter3=GENE_SYMBOL&srchType=keyword&adv_kw_filter2=GENE_SYMBOL&group1=noValue&SearchRequest.Common.QueryText=FOXA1&adv_kw_filter1=GENE_SYMBOL&inventoried=1&adv_query_text3=&species=Homo+sapiens&adv_query_text2=&adv_query_text1=&paraTreeViewNode=null&adv_boolean3=AND&displayAdvSearchResults=&SearchRequest.Common.ResultsPerPage=25&adv_boolean2=AND&adv_boolean1=AND&chkBatchQueryText=false&kwfilter=GENE_SYMBOL&SearchRequest.Common.PageNumber=1&formatType=default&isSL=N& ) |
| FOXA3 | Hepatocyte nuclear factor 3-gamma (HNF-3G) (Forkhead box protein A3) | [Hs00270130_m1](https://products.appliedbiosystems.com/ab/en/US/adirect/ab?cmd=ABAssayDetailDisplay&assayID=Hs00270130_m1&Fs=y&adv_phrase3=EXACT&adv_phrase2=EXACT&adv_phrase1=EXACT&assayType=ge&catID=601267&SearchRequest.Common.SortSpec=&searchValue=null&MFCSpeciesType=null&searchBy=null&adv_kw_filter3=all&srchType=keyword&adv_kw_filter2=all&group1=noValue&SearchRequest.Common.QueryText=FOXA3&adv_kw_filter1=all&inventoried=1&adv_query_text3=&species=Homo+sapiens&adv_query_text2=&adv_query_text1=&paraTreeViewNode=null&adv_boolean3=AND&displayAdvSearchResults=&SearchRequest.Common.ResultsPerPage=25&adv_boolean2=AND&adv_boolean1=AND&chkBatchQueryText=false&kwfilter=all&SearchRequest.Common.PageNumber=1&formatType=default&isSL=N& ) |
| FOXO3A | Forkhead box protein O3A | [Hs00818121_m1](https://products.appliedbiosystems.com/ab/en/US/adirect/ab?cmd=ABAssayDetailDisplay&assayID=Hs00818121_m1&Fs=y&adv_phrase3=EXACT&adv_phrase2=EXACT&adv_phrase1=EXACT&assayType=ge&catID=601267&SearchRequest.Common.SortSpec=&searchValue=null&MFCSpeciesType=null&searchBy=null&adv_kw_filter3=all&srchType=keyword&adv_kw_filter2=all&group1=noValue&SearchRequest.Common.QueryText=FOXO3A&adv_kw_filter1=all&inventoried=1&adv_query_text3=&species=Homo+sapiens&adv_query_text2=&adv_query_text1=&paraTreeViewNode=null&adv_boolean3=AND&displayAdvSearchResults=&SearchRequest.Common.ResultsPerPage=25&adv_boolean2=AND&adv_boolean1=AND&chkBatchQueryText=false&kwfilter=all&SearchRequest.Common.PageNumber=1&formatType=default&isSL=N& ) |
| FYN | Proto-oncogene tyrosine-protein kinase Fyn | Hs00176628_m1 |
| GPER | G Protein Coupled Estrogen Receptor 1 (GPR30) | [Hs00173506_m1](https://products.appliedbiosystems.com/ab/en/US/adirect/ab?cmd=ABAssayDetailDisplay&assayID=Hs00173506_m1&Fs=y&adv_phrase3=EXACT&adv_phrase2=EXACT&adv_phrase1=EXACT&assayType=ge&catID=601267&SearchRequest.Common.SortSpec=&searchValue=null&MFCSpeciesType=null&searchBy=null&adv_kw_filter3=GENE_SYMBOL&srchType=keyword&adv_kw_filter2=GENE_SYMBOL&group1=noValue&SearchRequest.Common.QueryText=GPER&adv_kw_filter1=GENE_SYMBOL&inventoried=*&adv_query_text3=&species=Homo+sapiens&adv_query_text2=&adv_query_text1=&paraTreeViewNode=null&adv_boolean3=AND&displayAdvSearchResults=&SearchRequest.Common.ResultsPerPage=25&adv_boolean2=AND&adv_boolean1=AND&chkBatchQueryText=false&kwfilter=GENE_SYMBOL&SearchRequest.Common.PageNumber=1&formatType=default&isSL=N& ) |
| GRB2 | Growth Factor Receptor Bound Protein 2 | [Hs00157817_m1](https://products.appliedbiosystems.com/ab/en/US/adirect/ab?cmd=ABAssayDetailDisplay&assayID=Hs00157817_m1&Fs=y&adv_phrase3=EXACT&adv_phrase2=EXACT&adv_phrase1=EXACT&assayType=ge&catID=601267&SearchRequest.Common.SortSpec=&searchValue=null&MFCSpeciesType=null&searchBy=null&adv_kw_filter3=all&srchType=keyword&adv_kw_filter2=all&group1=noValue&SearchRequest.Common.QueryText=GRB2&adv_kw_filter1=all&inventoried=1&adv_query_text3=&species=Homo+sapiens&adv_query_text2=&adv_query_text1=&paraTreeViewNode=null&adv_boolean3=AND&displayAdvSearchResults=&SearchRequest.Common.ResultsPerPage=25&adv_boolean2=AND&adv_boolean1=AND&chkBatchQueryText=false&kwfilter=all&SearchRequest.Common.PageNumber=1&formatType=default&isSL=N& ) |
| GRB7 | Growth Factor Receptor Bound Protein 7 | Hs00180450_m1 |
| GSTM1 | Gluthione S-Transferase | Hs02341469_m1 |
| IGF1R | Insulin-like growth factor 1 receptor precursor | [Hs00609566_m1](https://products.appliedbiosystems.com/ab/en/US/adirect/ab?cmd=ABAssayDetailDisplay&assayID=Hs00609566_m1&Fs=y&adv_phrase3=EXACT&adv_phrase2=EXACT&adv_phrase1=EXACT&assayType=ge&catID=601267&SearchRequest.Common.SortSpec=&searchValue=null&MFCSpeciesType=null&searchBy=null&adv_kw_filter3=all&srchType=keyword&adv_kw_filter2=all&group1=noValue&SearchRequest.Common.QueryText=IGF1R&adv_kw_filter1=all&inventoried=1&adv_query_text3=&species=Homo+sapiens&adv_query_text2=&adv_query_text1=&paraTreeViewNode=null&adv_boolean3=AND&displayAdvSearchResults=&SearchRequest.Common.ResultsPerPage=25&adv_boolean2=AND&adv_boolean1=AND&chkBatchQueryText=false&kwfilter=all&SearchRequest.Common.PageNumber=1&formatType=default&isSL=N& ) |
| IRAK1 | Interleukin-1 receptor-associated kinase 1 | Hs00155570_m1 |
| JUN | Transcription factor AP-1 (Activator protein 1) (AP1) (Proto-oncogene c-jun) | [Hs99999141_s1](https://products.appliedbiosystems.com/ab/en/US/adirect/ab?cmd=ABAssayDetailDisplay&assayID=Hs99999141_s1&Fs=y&adv_phrase3=EXACT&adv_phrase2=EXACT&adv_phrase1=EXACT&assayType=ge&catID=601267&SearchRequest.Common.SortSpec=&searchValue=null&MFCSpeciesType=null&searchBy=null&adv_kw_filter3=GENE_SYMBOL&srchType=keyword&adv_kw_filter2=GENE_SYMBOL&group1=noValue&SearchRequest.Common.QueryText=JUN&adv_kw_filter1=GENE_SYMBOL&inventoried=*&adv_query_text3=&species=Homo+sapiens&adv_query_text2=&adv_query_text1=&paraTreeViewNode=null&adv_boolean3=AND&displayAdvSearchResults=&SearchRequest.Common.ResultsPerPage=25&adv_boolean2=AND&adv_boolean1=AND&chkBatchQueryText=false&kwfilter=GENE_SYMBOL&SearchRequest.Common.PageNumber=1&formatType=default&isSL=N& ) |
| KDR | Vascular endothelial growth factor receptor 2 precursor | Hs00176676_m1 |
| KIT | Proto-oncogene tyrosine-protein kinase Kit) | Hs00174029_m1 |
| KRAS | v-Ki-ras2 Kirsten rat sarcoma viral oncogene homolog | [Hs00364282_m1](https://products.appliedbiosystems.com/ab/en/US/adirect/ab?cmd=ABAssayDetailDisplay&assayID=Hs00364282_m1&Fs=y&adv_phrase3=EXACT&adv_phrase2=EXACT&adv_phrase1=EXACT&assayType=ge&catID=601267&SearchRequest.Common.SortSpec=&searchValue=null&MFCSpeciesType=null&searchBy=null&adv_kw_filter3=all&srchType=keyword&adv_kw_filter2=all&group1=noValue&SearchRequest.Common.QueryText=KRAS&adv_kw_filter1=all&inventoried=1&adv_query_text3=&species=Homo+sapiens&adv_query_text2=&adv_query_text1=&paraTreeViewNode=null&adv_boolean3=AND&displayAdvSearchResults=&SearchRequest.Common.ResultsPerPage=25&adv_boolean2=AND&adv_boolean1=AND&chkBatchQueryText=false&kwfilter=all&SearchRequest.Common.PageNumber=1&formatType=default&isSL=N& ) |
| MAP3K4 | Mitogen-activated protein kinase kinase kinase 4 | Hs00245968_m1 |
| MAPK1 | Mitogen-activated protein kinase 1, Extracellular signal-regulated kinase 2 (ERK-2) | [Hs00177066_m1](https://products.appliedbiosystems.com/ab/en/US/adirect/ab?cmd=ABAssayDetailDisplay&assayID=Hs00177066_m1&Fs=y&adv_phrase3=EXACT&adv_phrase2=EXACT&adv_phrase1=EXACT&assayType=ge&catID=601267&SearchRequest.Common.SortSpec=&searchValue=null&MFCSpeciesType=null&searchBy=null&adv_kw_filter3=all&srchType=keyword&adv_kw_filter2=all&group1=noValue&SearchRequest.Common.QueryText=ERK2&adv_kw_filter1=all&inventoried=1&adv_query_text3=&adv_query_text2=&adv_query_text1=&paraTreeViewNode=null&adv_boolean3=AND&displayAdvSearchResults=&SearchRequest.Common.ResultsPerPage=25&adv_boolean2=AND&adv_boolean1=AND&chkBatchQueryText=false&kwfilter=all&SearchRequest.Common.PageNumber=1&formatType=default&isSL=N& ) |
| MAPK3 | Mitogen-activated protein kinase 3, Extracellular signal-regulated kinase 1 (ERK-1) | [Hs00385075_m1](https://products.appliedbiosystems.com/ab/en/US/adirect/ab?cmd=ABAssayDetailDisplay&assayID=Hs00385075_m1&Fs=y&adv_phrase3=EXACT&adv_phrase2=EXACT&adv_phrase1=EXACT&assayType=ge&catID=601267&SearchRequest.Common.SortSpec=&searchValue=null&MFCSpeciesType=null&searchBy=null&adv_kw_filter3=all&srchType=keyword&adv_kw_filter2=all&group1=noValue&SearchRequest.Common.QueryText=ERK1&adv_kw_filter1=all&inventoried=1&adv_query_text3=&adv_query_text2=&adv_query_text1=&paraTreeViewNode=null&adv_boolean3=AND&displayAdvSearchResults=&SearchRequest.Common.ResultsPerPage=25&adv_boolean2=AND&adv_boolean1=AND&chkBatchQueryText=false&kwfilter=all&SearchRequest.Common.PageNumber=1&formatType=default&isSL=N& ) |
| MET | Met proto-oncogene tyrosine kinase) | Hs00179845_m1 |
| MKI67 | antigen identified by monoclonal antibody Ki-67 | Hs00606991_m1 |
| MMP10 | Matrix Metalloproteinase 10 | Hs00233987_m1 |
| MMP11 | Matrix Metalloproteinase 11 | Hs00171829_m1 |
| MMP3 | Matrix Metalloproteinase 9 | [Hs00968308_m1](https://products.appliedbiosystems.com/ab/en/US/adirect/ab?cmd=ABAssayDetailDisplay&assayID=Hs00968308_m1&Fs=y&adv_phrase3=EXACT&adv_phrase2=EXACT&adv_phrase1=EXACT&assayType=ge&catID=601267&SearchRequest.Common.SortSpec=&searchValue=null&MFCSpeciesType=null&searchBy=null&adv_kw_filter3=all&srchType=keyword&adv_kw_filter2=all&group1=noValue&SearchRequest.Common.QueryText=MMP3&adv_kw_filter1=all&inventoried=1&adv_query_text3=&species=Homo+sapiens&adv_query_text2=&adv_query_text1=&paraTreeViewNode=null&adv_boolean3=AND&displayAdvSearchResults=&SearchRequest.Common.ResultsPerPage=25&adv_boolean2=AND&adv_boolean1=AND&chkBatchQueryText=false&kwfilter=all&SearchRequest.Common.PageNumber=1&formatType=default&isSL=N& ) |
| MMP9 | Matrix Metalloproteinase 9 | Hs00234579_m1 |
| MRPL19 | Mitochondrial Ribosomal Protein L19 | Hs00608519_m1 |
| MUC1 / CA-15 3 | Mucin-1 precursor | [Hs00410317_m1](https://products.appliedbiosystems.com/ab/en/US/adirect/ab?cmd=ABAssayDetailDisplay&assayID=Hs00410317_m1&Fs=y&adv_phrase3=EXACT&adv_phrase2=EXACT&adv_phrase1=EXACT&assayType=ge&catID=601267&SearchRequest.Common.SortSpec=&searchValue=null&MFCSpeciesType=null&searchBy=null&adv_kw_filter3=all&srchType=keyword&adv_kw_filter2=all&group1=noValue&SearchRequest.Common.QueryText=MUC1&adv_kw_filter1=all&inventoried=1&adv_query_text3=&species=Homo+sapiens&adv_query_text2=&adv_query_text1=&paraTreeViewNode=null&adv_boolean3=AND&displayAdvSearchResults=&SearchRequest.Common.ResultsPerPage=25&adv_boolean2=AND&adv_boolean1=AND&chkBatchQueryText=false&kwfilter=all&SearchRequest.Common.PageNumber=1&formatType=default&isSL=N& ) |
| MXD3 | Ras-related protein Rab-24. | [Hs00361007_m1](https://products.appliedbiosystems.com/ab/en/US/adirect/ab?cmd=ABAssayDetailDisplay&assayID=Hs00361007_m1&Fs=y&adv_phrase3=EXACT&adv_phrase2=EXACT&adv_phrase1=EXACT&assayType=ge&catID=601267&SearchRequest.Common.SortSpec=&searchValue=null&MFCSpeciesType=null&searchBy=null&adv_kw_filter3=all&srchType=keyword&adv_kw_filter2=all&group1=noValue&SearchRequest.Common.QueryText=MXD3&adv_kw_filter1=all&inventoried=1&adv_query_text3=&species=Homo+sapiens&adv_query_text2=&adv_query_text1=&paraTreeViewNode=null&adv_boolean3=AND&displayAdvSearchResults=&SearchRequest.Common.ResultsPerPage=25&adv_boolean2=AND&adv_boolean1=AND&chkBatchQueryText=false&kwfilter=all&SearchRequest.Common.PageNumber=1&formatType=default&isSL=N& ) |
| MYBL2 | Myeloblastosis Oncogene-like 2 | Hs00231158_m1 |
| NCOA4 | Nuclear receptor coactivator 4 | [Hs00428328_m1](https://products.appliedbiosystems.com/ab/en/US/adirect/ab?cmd=ABAssayDetailDisplay&assayID=Hs00428328_m1&Fs=y&adv_phrase3=EXACT&adv_phrase2=EXACT&adv_phrase1=EXACT&assayType=ge&catID=601267&SearchRequest.Common.SortSpec=&searchValue=null&MFCSpeciesType=null&searchBy=null&adv_kw_filter3=all&srchType=keyword&adv_kw_filter2=all&group1=noValue&SearchRequest.Common.QueryText=NCOA4&adv_kw_filter1=all&inventoried=1&adv_query_text3=&species=Homo+sapiens&adv_query_text2=&adv_query_text1=&paraTreeViewNode=null&adv_boolean3=AND&displayAdvSearchResults=&SearchRequest.Common.ResultsPerPage=25&adv_boolean2=AND&adv_boolean1=AND&chkBatchQueryText=false&kwfilter=all&SearchRequest.Common.PageNumber=1&formatType=default&isSL=N& ) |
| NCOR2 | Nuclear Receptor Corepressor 2 | Hs00196955_m1 |
| NFKB1 | Nuclear factor NF-kappa-B p105 subunit | [Hs00765730_m1](https://products.appliedbiosystems.com/ab/en/US/adirect/ab?cmd=ABAssayDetailDisplay&assayID=Hs00765730_m1&Fs=y&adv_phrase3=EXACT&adv_phrase2=EXACT&adv_phrase1=EXACT&assayType=ge&catID=601267&SearchRequest.Common.SortSpec=&searchValue=null&MFCSpeciesType=null&searchBy=null&adv_kw_filter3=GENE_SYMBOL&srchType=keyword&adv_kw_filter2=GENE_SYMBOL&group1=noValue&SearchRequest.Common.QueryText=NFKB1&adv_kw_filter1=GENE_SYMBOL&inventoried=*&adv_query_text3=&species=Homo+sapiens&adv_query_text2=&adv_query_text1=&paraTreeViewNode=null&adv_boolean3=AND&displayAdvSearchResults=&SearchRequest.Common.ResultsPerPage=25&adv_boolean2=AND&adv_boolean1=AND&chkBatchQueryText=false&kwfilter=GENE_SYMBOL&SearchRequest.Common.PageNumber=1&formatType=default&isSL=N& ) |
| PCAF | Histone acetyltransferase PCAF | [Hs00187332_m1](https://products.appliedbiosystems.com/ab/en/US/adirect/ab?cmd=ABAssayDetailDisplay&assayID=Hs00187332_m1&Fs=y&adv_phrase3=EXACT&adv_phrase2=EXACT&adv_phrase1=EXACT&assayType=ge&catID=601267&SearchRequest.Common.SortSpec=&searchValue=null&MFCSpeciesType=null&searchBy=null&adv_kw_filter3=GENE_SYMBOL&srchType=keyword&adv_kw_filter2=GENE_SYMBOL&group1=noValue&SearchRequest.Common.QueryText=PCAF&adv_kw_filter1=GENE_SYMBOL&inventoried=*&adv_query_text3=&species=Homo+sapiens&adv_query_text2=&adv_query_text1=&paraTreeViewNode=null&adv_boolean3=AND&displayAdvSearchResults=&SearchRequest.Common.ResultsPerPage=25&adv_boolean2=AND&adv_boolean1=AND&chkBatchQueryText=false&kwfilter=GENE_SYMBOL&SearchRequest.Common.PageNumber=1&formatType=default&isSL=N& ) |
| PDCD4 | Programmed cell death protein 4 | [Hs00377253_m1](https://products.appliedbiosystems.com/ab/en/US/adirect/ab?cmd=ABAssayDetailDisplay&assayID=Hs00377253_m1&Fs=y&adv_phrase3=EXACT&adv_phrase2=EXACT&adv_phrase1=EXACT&assayType=ge&catID=601267&SearchRequest.Common.SortSpec=&searchValue=null&MFCSpeciesType=null&searchBy=null&adv_kw_filter3=all&srchType=keyword&adv_kw_filter2=all&group1=noValue&SearchRequest.Common.QueryText=PDCD4&adv_kw_filter1=all&inventoried=1&adv_query_text3=&species=Homo+sapiens&adv_query_text2=&adv_query_text1=&paraTreeViewNode=null&adv_boolean3=AND&displayAdvSearchResults=&SearchRequest.Common.ResultsPerPage=25&adv_boolean2=AND&adv_boolean1=AND&chkBatchQueryText=false&kwfilter=all&SearchRequest.Common.PageNumber=1&formatType=default&isSL=N& ) |
| PDGFRA | Alpha platelet-derived growth factor receptor precursor | Hs00183486_m1 |
| PELP1 | Proline, glutamate and leucine rich protein 1 | [Hs00300396_m1](https://products.appliedbiosystems.com/ab/en/US/adirect/ab?cmd=ABAssayDetailDisplay&assayID=Hs00300396_m1&Fs=y&adv_phrase3=EXACT&adv_phrase2=EXACT&adv_phrase1=EXACT&assayType=ge&catID=601267&SearchRequest.Common.SortSpec=&searchValue=null&MFCSpeciesType=null&searchBy=null&adv_kw_filter3=GENE_SYMBOL&srchType=keyword&adv_kw_filter2=GENE_SYMBOL&group1=noValue&SearchRequest.Common.QueryText=PELP1&adv_kw_filter1=GENE_SYMBOL&inventoried=*&adv_query_text3=&species=Homo+sapiens&adv_query_text2=&adv_query_text1=&paraTreeViewNode=null&adv_boolean3=AND&displayAdvSearchResults=&SearchRequest.Common.ResultsPerPage=25&adv_boolean2=AND&adv_boolean1=AND&chkBatchQueryText=false&kwfilter=GENE_SYMBOL&SearchRequest.Common.PageNumber=1&formatType=default&isSL=N& ) |
| PGR1 | Progesterone Receptor | Hs00172183_m1 |
| PHB2 | prohibitin 2, B-cell associated protein; repressor of estrogen receptor activity | [Hs00200720_m1](https://products.appliedbiosystems.com/ab/en/US/adirect/ab?cmd=ABAssayDetailDisplay&assayID=Hs00200720_m1&Fs=y&adv_phrase3=EXACT&adv_phrase2=EXACT&adv_phrase1=EXACT&assayType=ge&catID=601267&SearchRequest.Common.SortSpec=&searchValue=null&MFCSpeciesType=null&searchBy=null&adv_kw_filter3=GENE_SYMBOL&srchType=keyword&adv_kw_filter2=GENE_SYMBOL&group1=noValue&SearchRequest.Common.QueryText=PHB2&adv_kw_filter1=GENE_SYMBOL&adv_query_text3=&species=Homo+sapiens&adv_query_text2=&adv_query_text1=&paraTreeViewNode=null&adv_boolean3=AND&displayAdvSearchResults=&adv_boolean2=AND&adv_boolean1=AND&chkBatchQueryText=false&kwfilter=GENE_SYMBOL&SearchRequest.Common.PageNumber=1&formatType=default&isSL=N& ) |
| PPIA | Cyclophilin A | Hs99999904_m1 |
| PTEN | MMAC1 phosphatase and tensin homolog mutated in multiple advanced cancers 1 | [Hs00829813_s1](https://products.appliedbiosystems.com/ab/en/US/adirect/ab?cmd=ABAssayDetailDisplay&assayID=Hs00829813_s1&Fs=y&adv_phrase3=EXACT&adv_phrase2=EXACT&adv_phrase1=EXACT&assayType=ge&catID=601267&SearchRequest.Common.SortSpec=&searchValue=null&MFCSpeciesType=null&searchBy=null&adv_kw_filter3=all&srchType=keyword&adv_kw_filter2=all&group1=noValue&SearchRequest.Common.QueryText=PTEN&adv_kw_filter1=all&inventoried=1&adv_query_text3=&species=Homo+sapiens&adv_query_text2=&adv_query_text1=&paraTreeViewNode=null&adv_boolean3=AND&displayAdvSearchResults=&SearchRequest.Common.ResultsPerPage=25&adv_boolean2=AND&adv_boolean1=AND&chkBatchQueryText=false&kwfilter=all&SearchRequest.Common.PageNumber=1&formatType=default&isSL=N& ) |
| RAGE | MAPK/MAK/MRK overlapping kinase | [Hs00179504_m1](https://products.appliedbiosystems.com/ab/en/US/adirect/ab?cmd=ABAssayDetailDisplay&assayID=Hs00179504_m1&Fs=y&adv_phrase3=EXACT&adv_phrase2=EXACT&adv_phrase1=EXACT&assayType=ge&catID=601267&SearchRequest.Common.SortSpec=&searchValue=null&MFCSpeciesType=null&searchBy=null&adv_kw_filter3=all&srchType=keyword&adv_kw_filter2=all&group1=noValue&SearchRequest.Common.QueryText=RAGE&adv_kw_filter1=all&inventoried=1&adv_query_text3=&species=Homo+sapiens&adv_query_text2=&adv_query_text1=&paraTreeViewNode=null&adv_boolean3=AND&displayAdvSearchResults=&SearchRequest.Common.ResultsPerPage=25&adv_boolean2=AND&adv_boolean1=AND&chkBatchQueryText=false&kwfilter=all&SearchRequest.Common.PageNumber=1&formatType=default&isSL=N& ) |
| RARA | retinoic acid receptor, alpha | Hs00940446_m1 |
| SCUBE2 | Signal Peptide CUB-EGF-like domain containing protein | Hs00221277_m1 |
| SMAD7 | Mothers against decapentaplegic homolog 7 (SMAD 7) | [Hs00178696_m1](https://products.appliedbiosystems.com/ab/en/US/adirect/ab?cmd=ABAssayDetailDisplay&assayID=Hs00178696_m1&Fs=y&adv_phrase3=EXACT&adv_phrase2=EXACT&adv_phrase1=EXACT&assayType=ge&catID=601267&SearchRequest.Common.SortSpec=&searchValue=null&MFCSpeciesType=null&searchBy=null&adv_kw_filter3=all&srchType=keyword&adv_kw_filter2=all&group1=noValue&SearchRequest.Common.QueryText=SMAD7&adv_kw_filter1=all&inventoried=1&adv_query_text3=&species=Homo+sapiens&adv_query_text2=&adv_query_text1=&paraTreeViewNode=null&adv_boolean3=AND&displayAdvSearchResults=&SearchRequest.Common.ResultsPerPage=25&adv_boolean2=AND&adv_boolean1=AND&chkBatchQueryText=false&kwfilter=all&SearchRequest.Common.PageNumber=1&formatType=default&isSL=N& ) |
| SNCG | Gamma-synuclein (Persyn) (Breast cancer-specific gene 1 protein) | [Hs00268306_m1](https://products.appliedbiosystems.com/ab/en/US/adirect/ab?cmd=ABAssayDetailDisplay&assayID=Hs00268306_m1&Fs=y&adv_phrase3=EXACT&adv_phrase2=EXACT&adv_phrase1=EXACT&assayType=ge&catID=601267&SearchRequest.Common.SortSpec=&searchValue=null&MFCSpeciesType=null&searchBy=null&adv_kw_filter3=all&srchType=keyword&adv_kw_filter2=all&group1=noValue&SearchRequest.Common.QueryText=SNCG&adv_kw_filter1=all&inventoried=1&adv_query_text3=&species=Homo+sapiens&adv_query_text2=&adv_query_text1=&paraTreeViewNode=null&adv_boolean3=AND&displayAdvSearchResults=&SearchRequest.Common.ResultsPerPage=25&adv_boolean2=AND&adv_boolean1=AND&chkBatchQueryText=false&kwfilter=all&SearchRequest.Common.PageNumber=1&formatType=default&isSL=N& ) |
| SP1 | Sp1 transcription factor | [Hs00916521_m1](https://products.appliedbiosystems.com/ab/en/US/adirect/ab?cmd=ABAssayDetailDisplay&assayID=Hs00916521_m1&Fs=y&adv_phrase3=EXACT&adv_phrase2=EXACT&adv_phrase1=EXACT&assayType=ge&catID=601267&SearchRequest.Common.SortSpec=&searchValue=null&MFCSpeciesType=null&searchBy=null&adv_kw_filter3=GENE_SYMBOL&srchType=keyword&adv_kw_filter2=GENE_SYMBOL&group1=noValue&SearchRequest.Common.QueryText=SP1&adv_kw_filter1=GENE_SYMBOL&inventoried=*&adv_query_text3=&species=Homo+sapiens&adv_query_text2=&adv_query_text1=&paraTreeViewNode=null&adv_boolean3=AND&displayAdvSearchResults=&SearchRequest.Common.ResultsPerPage=25&adv_boolean2=AND&adv_boolean1=AND&chkBatchQueryText=false&kwfilter=GENE_SYMBOL&SearchRequest.Common.PageNumber=1&formatType=default&isSL=N& ) |
| SRA1 | Steroid receptor RNA activator 1 | [Hs00288796_m1](https://products.appliedbiosystems.com/ab/en/US/adirect/ab?cmd=ABAssayDetailDisplay&assayID=Hs00288796_m1&Fs=y&adv_phrase3=EXACT&adv_phrase2=EXACT&adv_phrase1=EXACT&assayType=ge&catID=601267&SearchRequest.Common.SortSpec=&searchValue=null&MFCSpeciesType=null&searchBy=null&adv_kw_filter3=GENE_SYMBOL&srchType=keyword&adv_kw_filter2=GENE_SYMBOL&group1=noValue&SearchRequest.Common.QueryText=SRA1&adv_kw_filter1=GENE_SYMBOL&inventoried=*&adv_query_text3=&species=Homo+sapiens&adv_query_text2=&adv_query_text1=&paraTreeViewNode=null&adv_boolean3=AND&displayAdvSearchResults=&SearchRequest.Common.ResultsPerPage=25&adv_boolean2=AND&adv_boolean1=AND&chkBatchQueryText=false&kwfilter=GENE_SYMBOL&SearchRequest.Common.PageNumber=1&formatType=default&isSL=N& ) |
| STARD3 | StAR-related lipid transfer (START) domain containing 3 | Hs00199052_m1 |
| STMN1 | Stathmin (Phosphoprotein p19) (pp19) (Oncoprotein 18) | Hs01027516_g1 |
| STYK1 | Tyrosine protein-kinase STYK1 | Hs00218290_m1 |
| TGFBR1 | transforming growth factor, beta receptor I (activin A receptor type II-like kinase, 53kDa) | Hs00610318_m1 |
| TGFBR2 | transforming growth factor, beta receptor II (70/80kDa) | Hs00559661_m1 |
| TOP2A | topoisomerase (DNA) II alpha 170kDa | Hs00172214_m1 |
| TPM1 | Tropomyosin-1 alpha chain | [Hs00165966_m1](https://products.appliedbiosystems.com/ab/en/US/adirect/ab?cmd=ABAssayDetailDisplay&assayID=Hs00165966_m1&Fs=y&adv_phrase3=EXACT&adv_phrase2=EXACT&adv_phrase1=EXACT&assayType=ge&catID=601267&SearchRequest.Common.SortSpec=&searchValue=null&MFCSpeciesType=null&searchBy=null&adv_kw_filter3=GENE_SYMBOL&srchType=keyword&adv_kw_filter2=GENE_SYMBOL&group1=noValue&SearchRequest.Common.QueryText=TPM1&adv_kw_filter1=GENE_SYMBOL&inventoried=1&adv_query_text3=&species=Homo+sapiens&adv_query_text2=&adv_query_text1=&paraTreeViewNode=null&adv_boolean3=AND&displayAdvSearchResults=&SearchRequest.Common.ResultsPerPage=25&adv_boolean2=AND&adv_boolean1=AND&chkBatchQueryText=false&kwfilter=GENE_SYMBOL&SearchRequest.Common.PageNumber=1&formatType=default&isSL=N& ) |
| VAV2 Oncogene | vav 2 guanine nucleotide exchange factor | Hs00610104_m1 |
| VEGFA | vascular endothelial growth factor A | Hs00900054_m1 |
| VIL2 / Ezrin | villin 2 (ezrin) | Hs00931646_m1 |
| XIST | X (inactive)-specific transcript | Hs01079824_m1 |
| ZNF43 | Zinc finger protein 43 | [Hs00268760_m1](https://products.appliedbiosystems.com/ab/en/US/adirect/ab?cmd=ABAssayDetailDisplay&assayID=Hs00268760_m1&Fs=y&adv_phrase3=EXACT&adv_phrase2=EXACT&adv_phrase1=EXACT&assayType=ge&catID=601267&SearchRequest.Common.SortSpec=&searchValue=null&MFCSpeciesType=null&searchBy=null&adv_kw_filter3=all&srchType=keyword&adv_kw_filter2=all&group1=noValue&SearchRequest.Common.QueryText=STK15&adv_kw_filter1=all&inventoried=1&adv_query_text3=&species=Homo+sapiens&adv_query_text2=&adv_query_text1=&paraTreeViewNode=null&adv_boolean3=AND&displayAdvSearchResults=&SearchRequest.Common.ResultsPerPage=25&adv_boolean2=AND&adv_boolean1=AND&chkBatchQueryText=false&kwfilter=all&SearchRequest.Common.PageNumber=1&formatType=default&isSL=N& ) |
